# International Commission on Trichinellosis: Recommendations for quality assurance in digestion testing programs for *Trichinella*

**DOI:** 10.1016/j.fawpar.2019.e00059

**Published:** 2019-06-05

**Authors:** Alvin A. Gajadhar, Karsten Noeckler, Pascal Boireau, Patrizia Rossi, Brad Scandrett, H. Ray Gamble

**Affiliations:** a Department of Veterinary Microbiology, Western College of Veterinary Medicine, University of Saskatchewan, 52 Campus Drive, Saskatoon, SK S7N 5B4, Canada; b Federal Institute for Risk Assessment, Head of Department for Biological Safety, Diedersdorfer Weg 1, 12277 Berlin, Germany; c Laboratory for Animal Health, ANSES, INRA, ENVA, Université Paris Est, Maisons Alfort, France; d European Union Reference Laboratory for Parasites, Department of Infectious Diseases, Italian National Institute of Health, Viale Regina Elena, 299 00161 Rome, Italy; e Centre for Food-borne and Animal Parasitology, Canadian Food Inspection Agency, 116 Veterinary Road, Saskatoon, SK S7N 2R3, Canada; f National Academy of Sciences, 500 Fifth Street NW, Washington, DC 20001, USA

**Keywords:** *Trichinella*, Testing, ICT recommendations, Quality assurance

## Abstract

Effective performance of digestion testing methods for *Trichinella*, and their use for the detection of infected animals and the prevention of human trichinellosis require system-wide incorporation of appropriate quality assurance (QA) practices. The recommendations of the International Commission on Trichinellosis (ICT) aim to facilitate reliable test results when laboratories operate within a quality management system (QMS) which includes: 1) a quality manual (or similar documentation of the QMS); 2) a validated test method with identified critical control points; 3) a training program; 4) procedures utilizing proficiency testing and other methods to confirm technical capability of analysts; 5) equipment calibration and maintenance; 6) standard operating procedures, related documentation and reporting; 7) procedures to enable continuous monitoring and improvements; and 8) regular internal and third party audits. The quality manual or similar documentation describes the QMS within a testing laboratory, and lists the QA policies and good laboratory practices. Quality assurance goals contained in such documentation are the foundation of an effective QA program and must be explicit, measurable, and expressed in terms of performance criteria for the test method based on purpose for testing. The digestion method is capable of consistently detecting *Trichinella* larvae in meat at a level of sensitivity that is recognized to be effective for use in controlling animal infection and preventing human disease. However, consistent performance of the assay is assured only when parameters of the test method have been defined, scientifically validated as fit for purpose, and used within an effective QMS. The essential components of a digestion assay, specifically the critical control points and minimum standards for test performance are described. Reliable proficiency samples and their appropriate use in a quality system are key factors for certifying and maintaining an effective testing laboratory, including qualifying, re-qualifying and disqualifying of analysts as appropriate. Thus recommendations are included for the preparation and use of proficiency samples in a *Trichinella* digestion testing laboratory. The minimum training requirements for analysts performing a quality assured digestion assay, as well as suggested requirements for the content of a training manual, are also outlined. Finally, these ICT recommendations include essential components and minimum standards for maintaining and achieving certification and maintenance of a laboratory performing digestion testing for *Trichinella*. The certification program for the laboratory, including qualifying analysts, may be administered by a National Reference Laboratory or an authorized third party certifying body, under the auspices of the appropriate competent authority.

## Highlights

• Quality assurance (QA) is required for reliable digestion testing for *Trichinella*.
• QA in *Trichinella* testing entails method validation, training and proficiency testing.
• QA is essential for *Trichinella* testing certification programs for food safety purposes.

## 1 Introduction

The quality assurance (QA) recommendations described in this paper are intended for *Trichinella* digestion testing programs for pigs and other animals in the food chain. These QA measures are based on the best scientific information currently available; they follow the principles of the International Organization for Standardization's (ISO) ISO/IEC 17025 and 17043 standards and complement or support modern guidelines set by other international organizations such as the World Organization for Animal Health (OIE) and the Codex Alimentarius Commission (CODEX). The recommendations were commissioned by the International Commission on Trichinellosis (ICT) and developed by an ICT Quality Assurance Committee (Appendix A, Supplemental Data) over a period of nearly three years, including four workshops that were held in Calgary, Canada (2009), Paris, France (2010), Changchun, China (2011) and Rome, Italy (2011). Appendix B (Supplemental Data) contains a list of QA terms and their definitions as relevant to *Trichinella* testing. The definitions were adapted from internationally recognized QA authorities which are referenced in this document. The definitions of QA terms should help in the understanding and application of these ICT recommendations, and facilitate their common use in testing programs. Similar information regarding these recommendations is also available on the ICT website (http:/www.trichinellosis.org/Guidelines.html). Advancements in QA knowledge and technology require continuous assessment and improvement of quality management systems (QMS). Wherever possible, these QA recommendations incorporate such a cycle.

The implementation, maintenance and enforcement of these minimum QA recommendations will incur additional costs for routine *Trichinella* testing, and the roles and responsibilities for implementation of these recommendations may have to be determined. For laboratories already accredited under the ISO/IEC 17025 standard, which does not prescribe such specific requirements, these recommendations can provide additional guidance for consideration when establishing this testing in-scope. The relevant public health or veterinary authority is ultimately responsible for determining minimum quality standards for *Trichinella* testing, such as proficiency testing (PT) and laboratory certification, and therefore should take a lead role in directing any implementation of these recommendations. When weighing the cost for proper QA and reliable test results against the impact of outbreaks of trichinellosis on public health and trade, the rationale for meeting these minimum recommendations is compelling.

## 2 Essential quality assurance standards for *Trichinella* digestion assays

Diagnosis and control of *Trichinella* infection in susceptible food animals and game are fundamental to ensuring consumer protection from exposure to this parasite. In this context, the effectiveness of any meat inspection system depends on the application of proper QA standards (Gajadhar et al., 2009). According to the ICT, digestion assays for the detection of *Trichinella* larvae in meat are required to meet internationally accepted standards which include scientifically derived validation data and a design that allows routine monitoring and documentation of critical control points (Gamble et al., 2000). For trade and food safety purposes, digestion assays are the only reliable procedures for the direct detection of *Trichinella* larvae in meat (Gamble et al., 2000; Forbes et al., 2003; World Organisation for Animal Health (OIE), 2018a, World Organisation for Animal Health (OIE), 2018b). These assays can be used on single or pooled muscle samples. The procedure involves enzymatic degradation of muscle fibres using acidified pepsin to release muscle larvae for subsequent isolation and identification (Nöckler and Kapel, 2007).

**Fig. 1:**
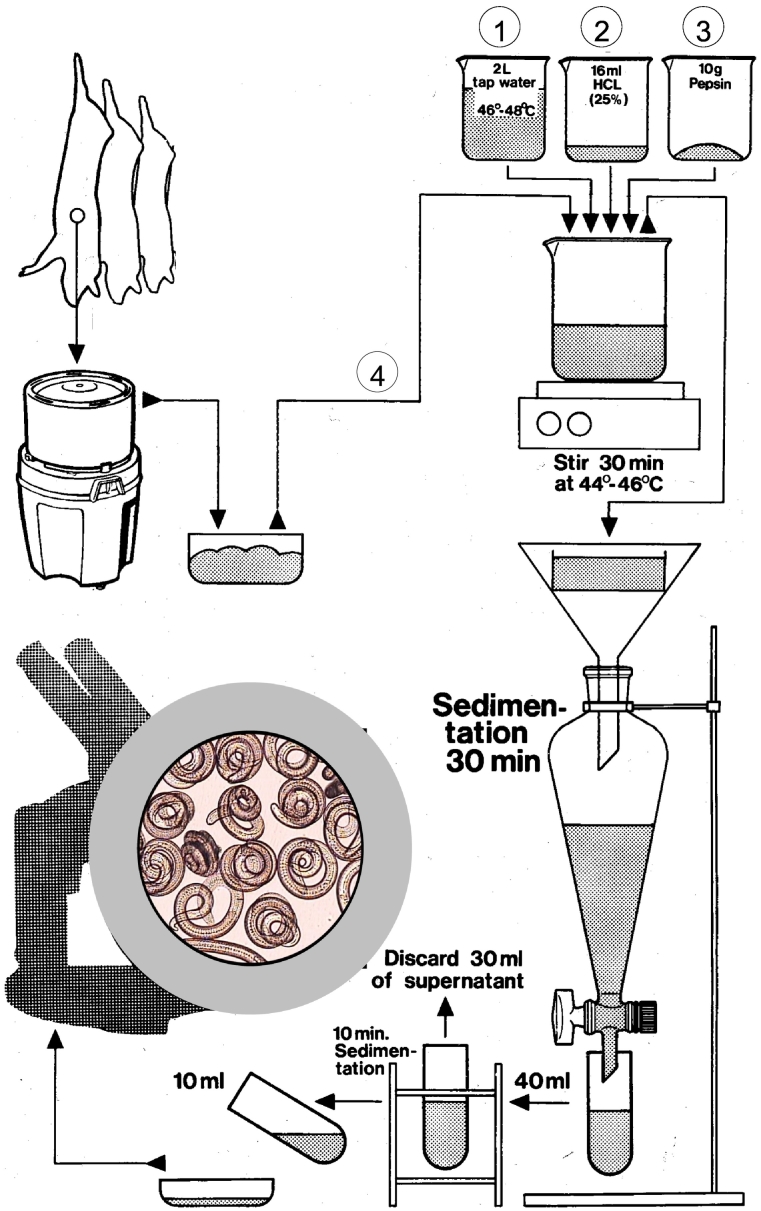
Diagram of a magnetic stirrer method for pooled sample digestion (steps labelled as 1–4 indicate the required sequential order for preparing the digest).

Of the several variations of the digestion assay, the magnetic stirrer method is the internationally accepted reference method (European Union, 2015; ISO, 2015a; OIE, 2018a; Webster et al., 2006) and is used as the focus of the QA standards for *Trichinella* digestion assays described in this document. Although a number of variations of this method currently exist, only a few have been adequately validated (Forbes and Gajadhar, 1999). An example of a magnetic stirrer method is shown in Fig. 1.

The objectives of this section are to:

a) Describe the components of the method which have the potential to influence the quality of *Trichinella* digestion testing;
b) List critical control points and minimum standards for performance of *Trichinella* digestion testing for meat inspection and surveillance;
c) Define the minimum QA measures for uniform performance of *Trichinella* digestion assays with a focus on the magnetic stirrer method.

### 2.1 Main components of quality assurance for *Trichinella* digestion testing

Artificial digestion is used for the *post-mortem* testing of carcasses for *Trichinella* infection for meat inspection, monitoring or surveillance of production animals (e.g. swine, horse, crocodile), game (e.g. wild boar, bear, walrus) and other wildlife (e.g. fox, raccoon dog) (Nöckler et al., 2000; Leclair et al., 2003; Larter et al., 2011).

Since digestion assays used for the detection of *Trichinella* larvae in meat do not include internal controls to monitor the effectiveness of the detection system, other tools for QA must be relied upon. The quality and accuracy of *Trichinella* testing is dependent on the proper performance of the digestion method, the appropriate sample collection based on the target species, adequate facilities, equipment and consumables, accurate verification of findings, and proper documentation of results (Gamble et al., 2000; Nöckler and Kapel, 2007). Thus, minimum QA standards should address the following main components:

a) Muscle sample collection and preparation for testing
b) Minimum requirements for equipment and consumables
c) Performance of the digestion assay
d) Verification of findings
e) Documentation

### 2.2 Critical control points and minimum standards

For meat inspection it is necessary to ensure a test sensitivity which allows detection of the lowest number of larvae that may cause clinical symptoms in humans (Dupouy-Camet and Bruschi, 2007; Nöckler and Kapel, 2007). Results from digestion test validation studies in pork show that a 1 g sample size reliably allows for the detection of ≥3 larvae per g (lpg) in muscle tissue whereas 3 and 5 g sample sizes can reliably detect ≥1.5 lpg and ≥1 lpg, respectively (Gamble, 1996; Forbes and Gajadhar, 1999).

Monitoring or surveillance via digestion assay may be used for demonstrating freedom of infection in a herd or region, documenting a very low occurrence of infection, or assessing prevalence in a population. The design of such sampling schemes should take into account factors known to affect test performance. For example, it has been reported that the infection burden in wildlife (e.g. foxes) is low, therefore larger-sized samples are used to improve sensitivity (Malakauskas et al., 2007). Similar adjustments may also be necessary to compensate for the lower digestibility of wildlife samples relative to that of pork diaphragm, resulting in a lower relative recovery of larvae (Kapel et al., 2005).

To ensure reliable test performance for the required detection sensitivity, minimum standards as described below, are recommended for sample collection and preparation, equipment and consumables, assay performance, results verification, and documentation.

#### 2.2.1 Sample collection and preparation for testing

In order to obtain the desired sensitivity for *Trichinella* testing in domestic or wild animals, an appropriate size of sample should be collected from a predilection muscle of the target animal species. An overview of predilection muscles for selected domestic and wild animals required for collection and examination by digestion assay is provided in Table 1. Muscle samples taken from the carcass for digestion testing should be at least twice the weight required for examination to allow for trimming of non-digestible tissues. For inspection of individual food animal carcasses for public health purposes, the sample size to be tested should be determined by the relevant competent authority based on scientific knowledge of test sensitivity and the reason for testing.

**Table 1:** Predilection muscles for select animal species that are recommended for digestion testing for *Trichinella* (Gamble et al., 2000; Kapel et al., 2003; Kapel et al., 2005; Larter et al., 2011; Leclair et al., 2003; Nöckler and Kapel, 2007).

Animal species	Predilection muscles
Domestic pig (*Sus scrofa domesticus*)	Diaphragm, masseter, tongue
Horse (*Equus caballus*)	Diaphragm, masseter, tongue
Wild boar (*Sus scrofa*)	Diaphragm, foreleg, tongue
Dog (*Canis lupus familiaris*)	Diaphragm, masseter, tongue
Bear (*Ursus* spp.)	Diaphragm, masseter, tongue
Walrus (*Odobenus rosmarus*)	Tongue
Seal (*Halichoerus grypus*)	Diaphragm, intercostal, tongue
Crocodile (*Crocodylus niloticus*)	Intercostal, masseter, tongue
Fox (*Vulpes* spp.)	Diaphragm, foreleg, tongue
Raccoon dog (*Nyctereutes procyonoides*)	Diaphragm, foreleg, tongue

Muscle samples should be labelled upon collection and tested as soon as possible or stored under cool conditions (such as 2–8 °C) that slow decomposition but avoid freezing. Samples which cannot be examined for some time after collection (such as for wildlife surveillance) should be kept cool in labelled plastic bags until testing can be performed. Extended storage by freezing is possible, but freezing can impair digestion and result in a loss in recovery of larvae that are not freeze-resistant; sample weight of frozen samples should be increased to compensate for the reduction in test sensitivity.

Samples tested by digestion assay should be free from non-digestible fat, tendons, fascia, etc. If tongue tissue is used, non-digestible connective tissue should be removed before testing. Samples must conform to the minimum required weight after trimming. Muscle samples of insufficient weight, dehydrated, or lacking identification do not meet minimum quality requirements and should be rejected by the laboratory (OIE, 2018c).

The minimum individual sample size for testing by the magnetic stirrer method following removal of non-digestible tissues depends on the required level of sensitivity. If a level of detection of at least 1 lpg of meat is required, a minimum of 5 g is required for testing, regardless of the age and origin of animal (Gamble, 1996; Forbes and Gajadhar, 1999). The maximum sample weight in a digestion pool should not exceed 115 g for a 2 L volume of digestion fluid. For pools with a lower total muscle weight (e.g. 50 g) the digest fluid volume and ingredients may be adjusted accordingly.

#### 2.2.2 Minimum requirements for equipment and consumables

All equipment used for the digestion assay must be properly cleaned prior to testing in order to avoid cross contamination. Table 2 contains a list of equipment and consumables that are required for *Trichinella* testing.

**Table 2:** Minimum requirement for equipment and consumables for quality assured *Trichinella* digestion testing.

Consumable supplies	Equipment
Labelled collection trays or plastic bags for samples	Knives, scissors and forceps for cutting samples and removing non-digestible tissue
Aluminum foil, parafilm or lids to cover the top of the glass beaker	Calibrated scale for weighing samples and/or pepsin (accurate to 0.1 g)
Tubes or measuring cylinders (50 or 100 ml plastic or glass)	Blender with a sharp chopping blade (regularly inspected and/or exchanged). The blender bowl should be made of acid resistant material (glass or stainless steel).
Petri dishes gridded with squares of 1 cm maximum dimension, or larval counting basin for trichinoscope (180 × 40 mm) marked off into squares	Magnetic stirrer with an adjustable heating plate
Pipettes (1, 10 and 25 ml)	Thermometer (accurate to 0.5–1.0 °C, 1 to 100 °C)
Tap water heated to 46 to 48 °C	Teflon-coated stir bar (5 cm long)
Hydrochloric acid (concentrated stock such as 25% or 37%)	Glass beakers (minimum 3 L capacity)
Pepsin powder or granular [1: 10,000 NF (US National Formulary), 1: 12,500 BP (British Pharmacopoeia), 2000 FIP (Fédération international de pharmacie)], or liquid pepsin (660 European Pharmacopoeia units/ml)	Glass or plastic funnel (approx. 15 cm or larger)
Ethanol (70–90% ethyl alcohol)	Sieve made of brass or stainless steel, mesh size approx. 180–200 μm (approx. 10 cm or larger)
Small vials for collection of recovered larvae	Conical glass separatory funnels (minimum 2.5 L capacity) preferably with Teflon safety plugs
	Stereo-microscope with adjustable sub-stage transmitted light source, or trichinoscope with a horizontal table, capable of minimum 10–20 X magnification. Image capture and storage capability (camera) recommended but not required to document suspect results.

Teflonware or plasticware should not be used for beakers or separatory funnels since a rough surface and electrostatic charge may contribute to larval adherence to the inner surface of the equipment. Calibration or verification as appropriate should be performed at least once a year for all instruments used for measurements, i.e. scale, thermometers and pipettes and all equipment should be routinely maintained. Such controls for equipment are also a requirement for ISO/IEC 17025 accredited laboratories.

#### 2.2.3 Performance of the digestion assay

##### 2.2.3.1 Blending of muscle samples

Single or pooled meat samples should be chopped by a blender, grinder, or similar device (one which is easy to thoroughly clean) to increase the surface of the sample for enzymatic degradation. The blending procedure should be adjusted to maximize digestion efficiency (Gajadhar et al., 2009). The intensity of blending is dependent on both speed and time, and on the construction and maintenance of the blending device. Blending should be continued until no visible pieces of meat remain (usually 5–10 s at maximum speed). Too little blending may result in incomplete digestion, while too much blending could possibly damage any muscle larvae present in the samples (Gamble et al., 2000).

After blending, the chopped meat should be transferred to a 3 L glass beaker which contains the digest fluid (see Section 2.2.3.2 below). A small amount of digest fluid may be added to the meat in the blender (max. 100 ml per 100 g meat) to facilitate tissue homogenization and transfer of homogenate from the blender into the glass beaker (OIE, 2018a). To avoid larval loss due to adhering muscle tissue, the chopping blade and the blender bowl should be rinsed with a small quantity of digest fluid which should then be poured into the glass beaker.

##### 2.2.3.2 Preparation of the digest fluid

The most critical step is the sequence of mixing of the constituents of the digestive fluid. This sequence should be in the following order: 1) water, 2) hydrochloric acid (HCl) and 3) pepsin. This sequence will prevent degradation of pepsin which could occur if it is exposed to concentrated HCl. It is recommended that mixing of digest fluid take place under a fume hood to prevent exposure of analysts to acid fumes and/or pepsin dust. Initially, 16 ml of 25% HCl acid or an equivalent volume of 37% HCl (see Section 2.2.2 of this document) is added to a glass beaker containing 2 L of tap water which should be preheated to 46–48 °C. Care should be taken not to exceed this temperature, as higher temperatures could destroy any larvae present in the samples, or reduce the activity of the pepsin. Stock solutions of HCl are available in formulations other than 25% and the volume used must therefore be adjusted accordingly to achieve a final concentration of 0.2% (pH = 1–2) (max. pepsin activity pH = 1.5–1.6). The pH of the digest fluid can easily be checked using pH (litmus) paper. Following addition of the HCl, a magnetic stir bar is placed in the beaker, and the beaker placed on a preheated stir plate.

Ten grams of pepsin should then be added to the digest fluid. The pepsin used for the preparation of digest fluid must have the appropriate activity required for digestion. The activity is expressed either in ‘NF’ (US National Formulary), ‘BP’ (British Pharmacopoeia), ‘FIP’ (Fédération Internationale de Pharmacie) or ‘Ph. Eur.’ (European Pharmacopoeia) units (see Section 2.2.2 of this document). Storage conditions and shelf life of the pepsin should be displayed on the label of the storage container. The use of granular or liquid pepsin, rather than powder, is recommended to reduce the risk of aerosolisation and possible allergic reaction to analysts. The final concentration of pepsin in the digest fluid should be 0.5%. Up to twofold higher concentrations of pepsin may be used without harm to *Trichinella* larvae, but should only be considered when muscle samples do not digest adequately using standard conditions. The chopped meat is the final ingredient to be added to the digest (Fig. 1), after which stirring is commenced.

##### 2.2.3.3 Digestion of chopped meat in the glass beaker

A maximum ratio of 1:20 of meat to digest fluid in the glass beaker and a constant temperature of 44–46 °C should be used throughout the process to facilitate an efficient and rapid digestion. Beakers and fluid may be pre-heated (e.g. to 46–48 °C) to maintain an initial temperature within the required range. To maintain a constant temperature and decrease evaporation during digestion, the glass beaker should be covered with aluminum foil or similar material and the temperature should be regularly monitored with a thermometer. An electronic thermostat is recommended. Use of an incubator with glass doors can facilitate monitoring and maintaining the required digestion temperature. During stirring, the digest fluid must be stirred at a sufficiently high speed to create a deep vortex without splashing.

The time usually required for complete digestion of muscle is 30 min. In the case of muscle samples which are less digestible such as wildlife tissues (see Section 2.2.1), the digestion time can be increased but should typically not exceed 60 min in total (Kapel et al., 2005).

Time-temperature conditions below the recommended values could result in incomplete digestion of the muscle tissue. Conversely, overheating (>50 °C) or prolonged digestion times could result in the inactivation of pepsin or destruction and loss of larvae.

##### 2.2.3.4 Filtration of the digest fluid

Following digestion, the fluid should be poured carefully (to avoid overflow) through a sieve (see Section 2.2.2) into a separatory funnel. Sieves do not need to be calibrated since they are used to retain undigested debris and are not intended for measurement; however, sieves should be carefully cleaned after use (without scratching) to avoid altering the mesh size. Sieves must be free of debris prior to use to allow digest fluid to pass through.

After pouring the digest fluid into the separatory funnel, the glass beaker and the sieve should be rinsed with an additional volume of tap water (minimum of 100 ml) into the separatory funnel to avoid larval loss due to larvae adhering to the surfaces of the beaker or to residual tissue on the sieve.

The weight of any appreciable amount of undigested tissue remaining on the sieve should be determined by weighing the sieve on a scale that has been tared (zeroed) using a clean identical sieve. The digestion process is considered compliant if residual debris remaining on the sieve is minimal (i.e. ≤ 5% of starting sample weight) and consists primarily of non-digestible tissue (typically consisting of fascia and other connective tissue). If visible muscle tissue remains on the sieve, the whole procedure must be repeated using fresh muscle samples. If no more muscle tissue can be obtained from the same carcass or pool of carcasses, the undigested portion of muscle tissue from the sieve should be digested again using freshly prepared digest solution and examined in addition to the initial digest.

##### 2.2.3.5 Sedimentation of the digest fluid

The sedimentation step of the completed digest in the separatory funnel allows sufficient time for larvae to settle at the bottom of the fluid column for subsequent recovery and enumeration. Although not necessary, gentle tapping of the funnel wall (e.g. every 10 min) may facilitate the larvae settling to the bottom of the funnel.

Coiled live larvae sediment at a rate of about 1 cm per min. The digest fluid should remain undisturbed in the funnel for a minimum of 30 min. If the sedimentation time is <30 min not all larvae may have settled and may not be recovered in the collected sediment.

If muscle samples have been frozen prior to digestion, larvae of freeze-susceptible *Trichinella* species are likely to be dead. As dead larvae un-coil upon release from the muscle tissue, their sedimentation speed decreases. Therefore, sedimentation time for frozen muscle samples where dead larvae are expected should be extended for up to 60 min.

##### 2.2.3.6 Collection of the primary and secondary sediment

Following sedimentation in the separatory funnel, approximately 40 ml (or as prescribed by the particular validated method used) of the digest fluid (primary sediment) should be quickly dispensed (free-flowed) into a 50 ml tube. The stopcock of the separatory funnel must be fully opened to ensure that no larvae are trapped on the edge of the opening or fail to be flushed out due to low flow velocity. The primary sediment should be allowed to stand for 10 min so that larvae can again settle to the bottom. If the dispensed volume of the primary sediment is too small, larvae may remain in the digest fluid in the separatory funnel and will be lost. Conversely, there may be more debris if the volume of the primary sediment is too high.

After sedimentation for 10 min, 30 ml of the supernatant should be carefully withdrawn from the 50 ml tube by aspiration from the top, without disturbing the sediment, leaving a volume of 10 ml. This secondary sediment should be poured into a gridded Petri dish or larval counting basin. To remove larvae which may stick to the surface of the inner wall, the tube should be rinsed with 10 ml of water which is then added to the Petri dish or larval counting basin.

The collection of the primary and secondary sediment may be modified by using a double separatory funnel technique (Forbes and Gajadhar, 1999; Gajadhar and Forbes, 2002). In this method, approximately 125 ml of the digest fluid (primary sediment) from the first separatory funnel should be dispensed into a 500 ml separatory funnel and the volume is adjusted to 500 ml with tap water at room temperature. This mixture should be allowed to settle for an additional 10 min, after which a sample of 20 ml (secondary sediment) should be recovered directly into a gridded Petri dish for the identification of the larvae.

##### 2.2.3.7 Microscopic examination

The Petri dish or larval counting basin containing the secondary sediment should stand undisturbed on the examination stage of the stereomicrocope for at least 1 min to allow larvae to settle before microscopic examination. Following this initial settling, microscopic examination should be done as soon as possible. If the secondary sediment is kept for longer than 30 min before examination, it should be properly labelled and kept secure; since precipitates can accumulate as the solution cools, additional clarification as described below may be required at the time of examination.

Accurate focussing of the stereomicroscope on the lower layer of the sediment is crucial for the identification of larvae and should be established prior to microscopic examination of the test sample. This can be done by focussing the microscope to ensure that gridlines of the Petri dish or larval counting basin are easily visualised through the digest fluid. Because the gridlines are on the outer surface of the bottom of the plate, the focus must be adjusted slightly upwards to the inner surface of the plate to bring larvae into sharp focus.

The secondary sediment must be transparent enough to enable easy identification of any larvae. If not, the secondary sediment must be further clarified as follows: 1) transfer the secondary sediment and a tap water rinse of the Petri dish or larval counting basin into the tube, adding additional tap water to a total volume of approximately 40 ml; 2) allow to sediment for 10 min; 3) carefully withdraw the supernatant (see Section 2.2.3.6), leaving a volume of 10 ml. This sediment and a 10 ml tap water rinse of the tube should then be poured into the original gridded Petri dish or larval counting basin.

The final 20 ml of recovered sediment and rinse fluid is systematically examined grid by grid with a stereomicroscope or trichinoscope at a 10–20 X magnification. Any suspect *Trichinella* or other nematode findings should be verified by examination at 60–100 X magnification.

Suspect or positive *Trichinella* results from pooled samples must be traced back from the pool to the carcass of origin via digestion of progressively smaller numbers of pooled samples of increased sample size from the implicated carcasses. Pooled samples with negative results should be ruled out and those yielding positive results continue to be sampled and tested until digestion of tissue from an individual carcass demonstrates the source of the positive result.

Following performance of the digestion method, specific decontamination and cleaning procedures must be employed for all equipment (e.g. blender/grinder compartment, glassware and counting chambers) and laboratory table surfaces. *Trichinella* larvae are inactivated after contact with hot water (≥ 70 °C for at least 1–2 min). However, it is important to note that larvae requiring subsequent confirmation by microscopic or molecular assays should not be inactivated using hot water, as per the recommendations for genotyping on the ICT website (http:/www.trichinellosis.org/Guidelines.html) and in Pozio and Zarlenga (2019).

#### 2.2.4 Verification of findings

Knowledge of the basic morphological characteristics of *Trichinella* larvae, including dimensions, shape, and colour is required to verify findings. First stage larvae are approximately 0.7 to 1.1 mm in length and 0.03 mm in width. The most distinguishing feature of *Trichinella* larvae, not recognized by stereomicroscopy but by compound microscopy, is the stichosome, which consists of a series of discoid cells lining the oesophagus and occupying the anterior half of the body. Live *Trichinella* larvae may appear coiled (when cold) or motile (when warm), or C-shaped when dead (Gamble et al., 2000).

If positive or doubtful findings occur, the larvae should be transferred with a minimal volume of fluid (such as 5–10 ul) into a small vial (1–2 ml) filled with 70–90% ethyl alcohol (final concentration) for preservation as soon as possible and subsequent molecular identification (PCR) should be performed at a qualified reference laboratory. After attempting to transfer a single larva, the pipette tip used in the transfer should be examined under a stereomicroscope to ensure that the larva did not remain on the tip.

#### 2.2.5 Documentation

Each laboratory must have a system of adequate documentation which demonstrates that *Trichinella* testing was correctly performed according to appropriate QA standards, and facilitates adequate traceback (see Section 5).

A laboratory worksheet (Table 3; Annex D of the ISO 18743 Standard) should be used by analysts to record data for test reports and is therefore a critical document for quality audits and traceback investigations. Key components of the laboratory worksheet include sample tracking information, documentation that the method has been performed correctly by qualified personnel, documentation of problems and irregularities, and a written record of results (Gajadhar et al., 2009). Laboratory worksheets should be stored according to the requirements of the competent authority.

**Table 3:**
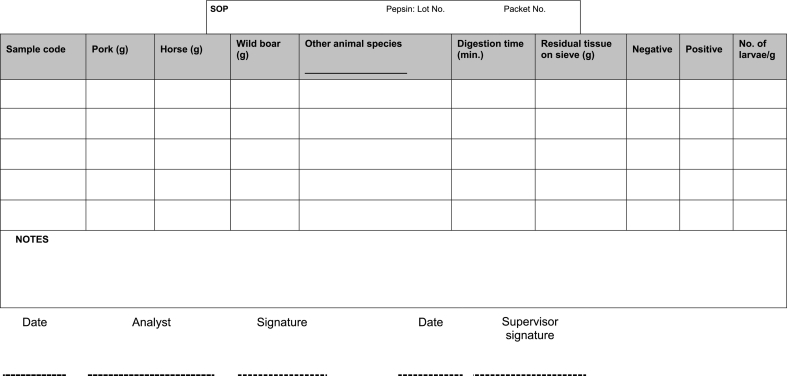
Example of a laboratory worksheet for recording data when testing pooled samples by *Trichinella* digestion assay.

## 3 Quality assurance in proficiency testing

### 3.1 Minimum requirements for production of proficiency samples

Proficiency samples enable the accurate assessment of test performance. Therefore, a reliable method to prepare proficiency samples containing known numbers of *Trichinella* larvae is an important component of a QA system for *Trichinella* digestion assays. To date, several methods for proficiency samples have been developed (Forbes et al., 1998; Marucci et al., 2009; Vallée et al., 2007). Although samples can be prepared by homogenizing muscle tissue with larvae in- situ, the numbers of larvae in such samples cannot be accurately quantified (Nöckler et al., 2009).

#### 3.1.1 Muscle tissue used in preparing proficiency samples

Source of muscle tissue: Muscle from the same host species and anatomical site routinely tested should be used (e.g. a laboratory which routinely tests pig diaphragm should use proficiency samples composed of tissue from pig diaphragm whenever feasible). Muscle with a low level of fat and fascia, or trimmed of such tissue, should be used for preparation of proficiency panels.

Weight and composition of muscle sample to be 'spiked' with *Trichinella* larvae: Muscle should be ground to facilitate preparation of 'meatballs' into which either encapsulated larvae (larvae still in the muscle capsule) or free larvae (larvae not in the muscle capsule) are inserted. For spiked samples using encapsulated larvae on agar plugs (Forbes et al., 1998) or using free larvae suspended in water, a minimum of 10 g of ground meat should be used to ensure that the spike is fully contained within the sample.

Additional muscle tissue required for use in completing the pool for digestion: Up to 100 g of additional muscle should be provided, free from contamination with *Trichinella* larvae, and meeting the same requirements as for the spiked sample.

#### 3.1.2 *Trichinella* larvae used in proficiency samples

Source of larvae: Laboratory animals such as mice, rats or guinea pigs may be used to propagate *Trichinella* spp. for proficiency samples. When determining a suitable animal model for this purpose, the species/genotype of *Trichinella* propagated and numbers of larvae required should be considered.

*Trichinella* species/genotype: *Trichinella* larvae from either encapsulating species that form capsules (cysts) in the host (*T. spiralis*, *T. nativa*, *T. britovi*, *T. murrelli*, *Trichinella*-T6, *T. nelsoni*, *Trichinella*-T8, *Trichinella*-T9, *T. patagoniensis*) or non-encapsulating species that do not form capsules in the host (*T. pseudospiralis*, *T. papuae*, *T. zimbabwensis*) may be used for the preparation of proficiency samples. However, larvae from non-encapsulating species recovered after digestion show survival times less than that of encapsulating species (European Union Reference Laboratory for Parasites, 2019). Therefore, encapsulating species are strongly recommended when available.

Use of encapsulated and free larvae: Encapsulated larvae or free larvae freshly released from capsules are used in proficiency samples. The use of encapsulated larvae is more labor intensive with respect to preparation and individual capsules must be assessed to ensure they contain only a single larva. However, encapsulated larvae are preferred not only because they are more resistant to environmental conditions, but because their use in proficiency testing (PT) helps to evaluate the ability of the digestion process used to release larvae from capsules for subsequent recovery and detection.

Methods to recover larvae from muscle for use in proficiency samples: The recovery of free larvae can be accomplished by the standard method of artificial digestion as described in Section 2 and by Marucci et al. (2009). Two procedures have been described for the harvesting of intact encapsulated *Trichinella* larvae:

a) The original method is based on filtration of blended infected rat muscle tissue. The blended muscle is mixed with phosphate-buffered saline and filtered through a double layer of tulle or gauze to yield a suspension of encysted larvae and fine muscle debris (Forbes et al., 1998).
b) A modified method generates larger numbers of encysted larvae for large-scale preparation of proficiency samples. The method incorporates an incomplete artificial digestion of muscle tissue from infected mice, followed by neutralisation of pepsin and HCl (Vallée et al., 2007).

Condition of larvae/capsules to be spiked: It is recommended that live *Trichinella* larvae be used for the preparation of proficiency samples. Death and degradation of larvae affects both their characteristic morphology and sedimentation in the funnel, and may cause false negative test results.

In *Trichinella* -free areas, risks of environmental or routine test sample contamination associated with the use of live larvae can be mitigated by following packaging and shipping procedures in accordance with international guidelines (IATA, 2019), and appropriate procedures for handling and containment of hazardous organisms. Additional mitigation measures include use of *Trichinella* species that have low or no infectivity for pigs.

Optimal storage conditions and shelf life for *Trichinella* larvae in proficiency samples should be determined for use in setting minimum recommendations. As the rate of degradation of larvae in intact capsules and larvae previously released from capsules may vary markedly, this should be determined separately for use in setting recommendations for storage conditions and shelf life.

#### 3.1.3 Preparation of proficiency samples

After recovery from muscles of an infected animal, encapsulated or free *Trichinella* larvae should be collected, counted and embedded (‘spiked’) into each sample. Specific methods to reliably prepare these samples have been described (Forbes et al., 1998; Marucci et al., 2009; Vallée et al., 2007). Any method used should be validated to assure it meets the requirements for its intended purpose, including the minimum standards described in these recommendations.

#### 3.1.4 Storage and transport of proficiency samples

Packaging (with/without vacuum-pack): Appropriate packaging must ensure no leakage of sample, including cysts or larvae. Vacuum packing can be used to prolong freshness of samples and larval survival.

Labelling: Sample labels must not contain the number of larvae in the spike. As a minimum, each sample should be labelled with a unique code that can be cross-indexed to a confidential master database maintained by the PT provider. The master database should contain details of sample production, including dates, spike numbers and intended recipients.

Storage conditions for samples: Proficiency samples should be stored at 5 °C ± 3 °C and shelf life limitations should be determined prior to distribution for testing. Samples made with either encapsulated or free larvae should not be frozen, unless the robustness of samples using freeze-tolerant species/genotypes of *Trichinella* has been validated.

Transportation of proficiency samples: Samples containing live larvae should be shipped/transported under bio-secure conditions for infectious material (UN 3373; OIE, 2018d) and under appropriate temperature conditions (5 °C ± 3 °C). Ideally, a probe to record the temperature during transport will facilitate monitoring of these conditions. Transport times should be minimised; receipt of samples within 48 h is recommended.

#### 3.1.5 Verification of proficiency sample integrity

Representative batch testing should be performed by the PT provider after samples have been prepared. Ideally, this should include initial testing of a representative group of samples prior to releasing the batch and final testing (to assess stability of the samples) following completion of the last sample in the field laboratory, or at the expiry date of the batch, whichever occurs first. Verification should include assessing the viability of larvae and the number of larvae contained in samples.

### 3.2 Proficiency testing panels for digestion assays

#### 3.2.1 Number of negative and positive samples per panel

The number of samples in a PT panel should be large enough to allow for variations in panel composition over time to ensure that panel composition is not predictable. It should include at least one negative sample and a minimum of two positive samples in order to evaluate the proficiency of a single analyst. PT panels consisting of a large number of samples should be avoided in routine use, as they do not provide significant additional information and may cause workflow interruptions in testing laboratories. However, each PT panel should consist of a minimum of three samples containing positive and negative samples as recommended above.

#### 3.2.2 Number of larvae per positive sample

Considering the sensitivity of the digestion assay (see Section 2), and the requirement to assess the technical ability of analysts to detect low numbers of larvae and the possibility of individual larvae being lost or damaged during sample production, it is recommended that positive samples within the PT panel should be spiked with 3–5 larvae, and at least one of these samples contain 3 larvae; numbers of larvae in samples should be verified as described in Section 3.1.5.

Spiked samples containing higher numbers of larvae can be useful for training, corrective actions, or validation of a digestion method, and may also be used as proficiency samples at the discretion of the PT provider or the certifying body.

#### 3.2.3 Frequency of testing panels

Each analyst should successfully complete at least one PT panel per year. Factors that may require increasing the frequency include:

Unsatisfactory results - a PT panel should be repeated immediately or other corrective actions taken.

Although national accreditation bodies adhering to the ISO/IEC 17025:2017 Standard (Section 7.7.2) require participation in PT and/or interlaboratory comparisons to assure the validity of test results, the specific frequency for these activities is not prescribed, but is typically performed a minimum of once per year. The recently developed ISO 22117:2019 standard provides additional specific requirements and guidance for organizing PT schemes by interlaboratory comparison for microbiological testing, including parasitology (ISO, 2019).

Ad-hoc local or national requirements – may be imposed by a competent authority for purposes such as ensuring that expertise is maintained in non-endemic regions.

### 3.3 Evaluation of proficiency testing results and implications for analyst and laboratory qualification for testing

#### 3.3.1 Evaluation of proficiency testing results

Proficiency samples are used to demonstrate test performance at an adequate level of sensitivity and for training and troubleshooting. Successful identification of samples containing a low number of larvae (3–5) indicates acceptable technical competence and an adequate analytical procedure. These samples represent low level infections which may be encountered in field testing.

Samples containing a higher number of larvae may be useful for training and for investigating problems within the testing system. High spike samples help to identify deviations from critical control points during recovery of larvae, problems in reading gridded plates (microscopic detection), and generally contain a wider variety of larval configurations than is usually seen with low spike samples.

Pass/fail criteria should be established to objectively measure the competence of an analyst in the performance of a digestion assay. Preparation, distribution and use of PT panels should be followed as recommended in other sections of this document. These criteria include stringently controlled production and distribution systems for PT to ensure that proficiency samples are not a source of error in evaluating technical proficiency.

##### 3.3.1.1 Acceptable recoveries of larvae from proficiency samples

Published data from PT schemes/programs in use in several countries indicate that recoveries of ≥75% of larvae in spiked samples are consistently achievable (Forbes and Gajadhar, 1999; Vallée et al., 2007). These data also show that laboratories with poor results improve rapidly when participating in a PT program, and that laboratories with good results generally employ more QA components in their testing systems, including use of a standardized test method. Although it is recommended that the correct identification of positive and negative samples in a PT panel be used for evaluating analyst performance, the reporting of results should include the number of larvae recovered from each sample. Such additional information facilitates documentation for continuous improvement and any future troubleshooting that may be required.

###### 3.3.1.1.1 Acceptable results for positive samples

For the recommended spike level of 3–5 larvae for positive samples in a PT panel, at least 1 larva should be recovered from each spiked sample within the panel.

**Pass**: Recovery of ≥1 larva
**Fail:** No larvae recovered*

*Reporting of additional larvae beyond the expected numbers may indicate false positive results and analyst competence should be investigated by review of performance data records and/or re-test.

###### 3.3.1.1.2 Acceptable results for negative samples

One or more negative samples should be included in each PT panel and should be varied to ensure that panel composition is not predictable. For example, a PT panel could contain either one or two negative samples.

**Pass**: Correct identification of a sample that does not contain larvae.

Fail*: Report of one or more larvae in a negative sample (false positive result).

* Failure of analyst in this case should be supported by confirmation of results, historical performance data or results of an immediate re-test as appropriate.

###### 3.3.1.1.3 Acceptable results for high-spiked samples

Samples spiked with high numbers of larvae are useful for training and troubleshooting. PT panels containing sample(s) with high spikes should also contain at least one low spiked sample, and all samples with 3–5 larvae should have acceptable recoveries to pass the panel. The actual number of larvae in high spiked samples and the evaluation of results should be determined according to the intended purpose, by the competent authority or designate (e.g. National Reference Laboratory).

#### 3.3.2 Proficiency requirements for qualification of analyst and laboratory

##### 3.3.2.1 Initial qualification of an analyst as competent to conduct digestion testing

For an analyst to be deemed qualified, PT panels should be passed as part of training exercises and on-site at the testing laboratory. If problems occur, the reference laboratory or PT provider can assist with troubleshooting to rule out non-technical causes, and may recommend re-training, re-testing or other actions as required. Follow-up actions can vary according to results, qualification status of the analyst, unforeseen factors affecting results and resources of the reference and testing laboratory. The comprehensive activities required for initial qualification are described in Section 4.

##### 3.3.2.2 Ongoing qualification of analysts

The purpose of ongoing evaluation is to demonstrate that qualified analysts in a testing laboratory continue to be competent in performing the assay. A qualified analyst must test at least one external PT panel at least once per year and meet the pass criteria as set out in these recommendations. The reference laboratory may provide solicited advice for troubleshooting problems and may provide non-scheduled proficiency samples under special arrangements. Any follow-up actions will similarly depend on factors indicated above.

##### 3.3.2.3 Initial and ongoing capacity of certified (or otherwise qualified) testing laboratories

The goal of PT is to assess, qualify and re-qualify individual analysts for performance of the artificial digestion method for *Trichinella*. A testing laboratory should have at least one qualified analyst as described in Sections 3.3.2.1 and 3.3.2.2 in order to achieve and maintain acceptable capacity for *Trichinella* digestion testing.

#### 3.3.3 Timelines for testing and reporting

Determination of testing schedules and reporting timelines is the responsibility of the PT provider and should take into account the shelf life of the proficiency samples.

##### 3.3.3.1 Testing laboratory

The testing laboratory should analyze proficiency samples and report back to the PT provider as stipulated by the PT provider and/or competent authority. The testing laboratory should immediately inform the PT provider and/or competent authority if analysts encounter any unexpected issues related to PT, and take appropriate remedial actions.

##### 3.3.3.2 Proficiency testing provider

A summary PT report (not identifying performance of individuals), which includes statistical analysis/comparison/trending of performance amongst all participating testing laboratories, is made available to participants within planned timeframes.

An official report on PT results of each individual participating laboratory should also be provided to each such laboratory by the PT provider.

##### 3.3.3.3 Competent authority

In case of failure in PT, the competent authority or designate (e.g. National Reference Laboratory) should evaluate and approve, or reject if not appropriate, the proposed corrective actions, and verify their timely implementation.

##### 3.3.3.4 Reference laboratory reports

###### 3.3.3.4.1 Record keeping

Requirements for record keeping are determined by the competent authority or designate. It is recommended that record keeping comply with internationally recognized QA standards such as ISO series documents.

###### 3.3.3.4.2 Testing laboratory

It is recommended that the record keeping activities of the testing laboratory associated with *Trichinella* digestion testing be based on the principles of ISO/IEC 17025. Formal accreditation to ISO/IEC 17025 is preferable but not essential.

###### 3.3.3.4.3 Proficiency testing provider

The record keeping system of the PT provider should be based on ISO/IEC 17025 requirements and ideally should also comply with ISO/IEC 17043 (ISO, 2017; ISO, 2010, respectively). Records of statistical analyses should follow ISO 13528 guidelines as appropriate (ISO, 2015b). An adequate documentation system should include records of PT planning, assessment of suitable homogeneity and stability of proficiency samples, sample preparation, PT panel configuration, verification testing, sample handling, packaging, labelling and distribution, data analysis, reporting and follow-up activities. A copy of each report generated by the PT provider should reside in the testing laboratory and with the PT provider.

## 4 Training and qualifying analysts for testing

All personnel performing any aspect of the artificial digestion assay for *Trichinella* for regulatory or food safety purposes should be trained to meet a set of minimum requirements. Training should take into account all QA measures in regulatory testing as described in Section 1, and should be performed in a laboratory which meets the QA standards as described in Section 5. The requirements for training as described here should follow the QA recommendations of test performance as described in Section 2, and incorporate the use of proficiency samples and periodic PT as described in Section 3. Training should be provided by qualified personnel and should be conducted in a bio-secure facility, as determined appropriate by the competent authority. The following minimal elements and necessary resources are required for training laboratory analysts to perform the digestion assay for *Trichinella*. Actual training should be consistent with requirements of national legislation and the national competent authority.

### 4.1 Training elements

Recommended components of a training program are described as follows and summarized in Table 4.

a) An introduction given to analysts should provide the historical setting and objectives of training, including the rationale for using the technology (pooled sample digestion testing) and the importance of the results of testing as they affect public health, trade and the economy. Relevant legislation, policies, guidelines and recommendations should be discussed. The potential consequences of a false negative result should be reviewed in light of public health consequences and the impact on the producers, packers and competent authorities.
b) Analysts should be provided with an overview of *Trichinella* including biology, epidemiology, control measures and public health implications of *Trichinella* infection in food animals and game meats. Emphasis should be placed on factors that will motivate analysts to understand the importance of their testing work. This includes: a description of the disease resulting from exposure to *Trichinella*, citing recent human cases; the social and economic impact (cost of testing, cost of outbreaks) of testing; and, the potential consequences of missing a positive carcass. Useful references for this part of the training include: Murrell and Pozio, 2011; Gamble et al., 2007; Gottstein et al., 2009; Pozio, 2007; and Veterinary Parasitology Special Issue: *Trichinella* and Trichinellosis, 2000.
c) Analysts should be given an overview of control programs and processes to prevent human exposure to *Trichinella* in meat from pigs and other food animals. They should also be provided with the theory of testing for *Trichinella*, focusing on the digestion method. Analysts should be informed of the requirements of qualifying and retaining qualification to test for purposes of food safety. Useful references include: Gajadhar et al., 2009; Nöckler and Kapel, 2007; and Alban et al., 2011.
d) Analysts should receive training on good laboratory practices. Quality assurance information on the components of a testing laboratory's QMS should be covered, including essential components of the digestion assay and its critical control points and minimum standards for test performance as described in Section 2. The importance of documentation and proper record keeping should be stressed. The training should also include a demonstration of the practical aspects of maintenance of all equipment and reagents used in the artificial digestion method.
e) The trainer(s) should provide a practical demonstration and detailed discussion of the artificial digestion method with special attention to critical control points as described in Section 2. The trainer should emphasize the importance of having an approved standard operating procedure (SOP) and other reference documents, such as a training manual and figures available during routine testing. Various pre- and post-training materials, such as these ICT recommendations and review articles may be useful aids for the trainer and analysts, in additional to other relevant references (European Union, 2015; Gamble et al., 2000, and OIE, 2018a).
f) The trainer(s) should provide an overview and demonstration on the operation and maintenance of the stereomicroscope and provide ample time for in- depth practical identification of *Trichinella* and other parasite larvae recovered from digestions. This part of the training should include a demonstration of sources of false positives, including other nematode larvae and artifacts. Photos should have a scale so as to familiarize analysts with the relative size of *Trichinella* larvae along with the characteristic spiral shape and movement of active larvae.
g) Analysts should perform adequately supervised practice of the artificial digestion/magnetic stirrer method using *Trichinella*-spiked samples until they are able to consistently recover and identify larvae.
h) The successful independent performance of a series of digestion tests is necessary for an analyst to be deemed qualified to test for the purposes of meeting regulatory requirements or food safety standards. For a trainee to be deemed a qualified analyst, at the time of training, a complete set of samples from a PT panel must be successfully tested as outlined in Section 3. The specific criteria for qualifying an analyst should be set by the testing laboratory or competent authority and available as an SOP or documented in the quality manual or elsewhere in the laboratory.

**Table 4:** Recommended topics for inclusion in a *Trichinella* testing training program.

Topic	Objectives	Key points
History	Demonstrate the importance of human cases both historically and at present	*Trichinella* is a public health hazard, worldwide. Analysts must be attentive to the possibility of finding *Trichinella* in any sample.
Life cycle of *Trichinella*	Describe the basic life history of the parasite, reproductive capacity index	The entire life cycle occurs in one host; infective larvae are found in nurse cells, which are modified muscle cells. Digestion frees the larvae from the capsule and that is what is observed in the test.
Phylogeny of *Trichinella*	Describe the species and genotypes relative to differences such as freeze resistance	It is not possible to differentiate species in the digestion test. If larvae are recovered, they need to be appropriately preserved for genotyping and traceback.
*Trichinella* morphology	Provide a detailed description of the anatomical structure of the parasite including the stichosome and the cuticle	This section must be sufficiently detailed so that analysts are able to accurately identify *Trichinella* and distinguish from artifacts and other nematode larvae. Discuss the use of microscopes – what is visible at the appropriate magnification – and the size of *Trichinella* relative to other nematodes. Photos of larvae in various shapes (coiled, moving, dead) should be used and the details from this part of the training should be reinforced during the practical sessions.
Epidemiology	Describe the domestic and wildlife cycles and the species and hosts involved; geographical distribution	Focus should be on at- risk species in the area to be covered by the testing. Differences should be described amongst pigs reared in biosecure (*Trichinella*-free) housing versus backyard and free-ranging pigs (at risk for *Trichinella* infection)
Clinical disease	Describe the clinical disease resulting from human exposure to infected meat and susceptibility to all genotypes of *Trichinella*	This section should include a discussion of the enteral and parenteral phases, the most common symptoms, diagnostic methods, and the treatment and outcome of infections. Some details of outbreaks should be given.
Detection in animals	Describe direct and indirect tests to detect infection – benefits and drawbacks to use; predilection sites in host species; note that *Trichinella*-infected animals do not show any signs of disease	The only tests currently suitable for protecting public health are direct tests – artificial digestion methods. Indirect tests may be used for surveillance. Provide some theory of the digestion method, including why *Trichinella* resist digestion when alive and the problems with recovering larvae following digestion if larvae are dead.
Prevention	Describe current programs to prevent infection in domestic pigs	Analysts should be made aware of national programs that are designed to prevent infection in food animals. Also, they should understand that free-range and outdoor pigs as well as wildlife are at risk and are much more likely to yield positive results.
Prophylaxis	Describe processes (cooking, freezing, curing) used to kill *Trichinella* in prepared meats	Specific guidelines are available for the commercial cooking, freezing and curing of pork products, when meat has not been otherwise proven free from *Trichinella*. Home cooking guidelines are also available. Curing may be insufficient to kill larvae if not done properly.
Laboratory safety	Describe requirements for biocontainment when working with infectious material	Biocontainment applies specifically to handling of proficiency samples, but trainees should be informed that any piece of meat might harbor *Trichinella* larvae until tested and determined free.

The following is provided as example outcome(s) that may occur from this PT during analyst training:

• analyst completes testing of the PT panel in a satisfactory manner and thus meets training requirement
• results on initial PT panel are unsatisfactory; trouble shooting and additional training is conducted and an additional PT panel is tested with satisfactory results
• trainee repeatedly fails to properly perform the test using PT panels and thus cannot fulfill analyst training requirement.

i) Discussion should be held with all candidate analysts regarding reporting requirements and other obligations as prescribed by the testing laboratory and the competent authority. Topics discussed should include procedures to be followed in the event of the identification of a positive sample. An outline of such procedures and other requirements should be included in an SOP and available at the testing laboratory.
j) Training should culminate in a short written examination or other form of evaluation to assess and document the knowledge acquired by the newly qualified analyst. This final session should include a review of the training, expectations regarding PT by the qualified analyst at the certified laboratory, and any feedback to the trainers for improvement of the training program.

### 4.2 Initial proficiency qualification, re-qualification, disqualification and re-training

Following training, and prior to performing testing for regulatory or food safety purposes, analysts should be required to demonstrate competency when performing the artificial digestion method in their laboratory. The specific requirements and criteria for analyst qualification, re-qualification, disqualification and re-training should be specified by the testing laboratory or competent authority, and appropriately documented at the laboratory. The criteria should incorporate the minimum recommendations for pass/fail as outlined in Section 3. Only a reasonable number of repeat tests should be allowed when failure on PT occurs, and the use of trouble-shooting and repeat training should be considered as corrective actions when appropriate. The following are examples for use in an ‘on-site’ qualification process and for additional monitoring and training requirements.

#### 4.2.1 On-site qualification – Initial proficiency samples

Following successful training, a new analyst should perform on-site PT at the testing laboratory as soon as possible, but no longer than three months from the initial training, and prior to conducting testing for regulatory or food safety purposes. Proficiency samples should be prepared and distributed by an agent of the competent authority using PT panels as described in Section 3. Proficiency samples tested on- site must be performed using the facilities and equipment which the analyst will use on a regular basis. Analysts must conduct PT independently without any assistance or interference from other persons.

In performing this initial on-site PT panel, the following outcomes may occur:

• the analyst completes testing in a satisfactory manner and is qualified to test for regulatory or food safety purposes
• the analyst fails the initial on-site PT panel; an additional panel is tested. If passed, the analyst is qualified to test for regulatory or food safety purposes.
• the second on-site PT panel is failed, and non-technical sources of error have been ruled out; the competent authority will determine if additional testing, training or analyst disqualification is appropriate

#### 4.2.2 Re-qualification

Analysts must be re-qualified at least once each year. Re-qualification is granted pending acceptable analysis of a PT panel as outlined above. In the event that PT results are not in compliance with the requirements of the competent authority, a re-test may be granted. Failure of an analyst to accurately analyze a second PT panel should result in disqualification and the individual should undergo re-training in accordance with the training plan outlined here.

#### 4.2.3 Re-training

The competent authority should determine a need for periodic updating of training for qualified analysts and specific training requirements for analysts who are disqualified based on failure of PT.

### 4.3 Recommended content of a training manual

#### 4.3.1 Introduction

The introduction should discuss the history and relevance of regulatory testing for *Trichinella* in food animal species and game meats intended for human consumption. It should include references to relevant legislation and outbreaks of *Trichinella*, which emphasize the importance of testing. It should describe the roles of qualified analysts performing the training and the roles of others in the testing chain, including the competent authority.

#### 4.3.2 Information about the parasite *Trichinella*

This section should include general information about *Trichinella* that will provide the qualified analyst with basic knowledge and an understanding of the importance of the testing which they perform. Recommended topics include: a) *Trichinella* life cycle and biology, b) *Trichinella* morphology, c) geographical distribution of species, and d) epidemiology in food animal species.

#### 4.3.3 Information about the disease

General information about the disease in humans (trichinellosis) should be provided as basic knowledge to the qualified analyst for an understanding of the importance of the testing that they perform. Recommended topics include: routes of exposure (outbreak examples), clinical features in humans, and diagnosis and treatment.

#### 4.3.4 Control methods

Control methods should provide basic information on how *Trichinella* is controlled – on the farm or production unit – and the options for preventing exposure of humans to infected meat (slaughter testing and post-slaughter processing). Recommended topics should include any of the following:

• Pre-harvest control – describe bio-secure production systems that reduce risk of exposure to *Trichinella* in domestic pigs
• Slaughter inspection – describe regulations governing slaughter inspection including the use of the pooled sample digestion method
• Processing – discuss further processing to inactivate *Trichinella* in ready-to-eat products
  o Cooking
  o Freezing
  o Curing
  o Irradiation
• Consumer preparation – Discuss home preparation of meats which have a risk of contamination with *Trichinella* larvae.

Resources containing useful information related to these subjects include:

Pre-harvest control: Codex, 2015; European Union, 2015; OIE, 2018b; Slaughter inspection: European Union, 2015; and Post-slaughter processing: European Union, 2015; USDA, 2018.

#### 4.3.5 Test methodologies

This section should include a detailed description of the methods to be used in testing for *Trichinella* in food animals and game meats intended for human consumption. Information for this purpose has been previously published (European Union, 2015; OIE, 2018a). Additional resources may be available from the local or national competent authority, including legislation and guidance documents.

#### 4.3.6 Quality assurance

This section should describe SOP's, a quality manual (or similar documentation), and record-keeping and reporting requirements, along with any other essential components of a laboratory QMS. A plan for recording and retaining results of all testing should be described which is consistent with recommendations found in Sections 2 and 5, and as required by the competent authority.

#### 4.3.7 Morphological identification of the parasite

This section should provide extensive and detailed information on the accurate identification of *Trichinella* in the digestion test. Both written descriptions and photographs should be used, including a scale of size. Side- by- side comparisons of *Trichinella* larvae with other parasite larvae are instructive. Subjects to be covered in this section include: a) photos of *Trichinella* – live(coiled, uncoiled and motile), dead(C-shaped); b) other parasite larvae that may be found in tissue digests; c) artifacts – pollen, bubbles, hairs, etc. Resources for these images may be found in a variety of places, including the internet.

#### 4.3.8 Proficiency testing

The quality of analyst performance requires periodic assessment, which is accomplished, in part, by the use of proficiency panels (see OIE - http://www.oie.int/our-scientific-expertise/reference-laboratories/proficiency-testing/). A description of the requirements and procedures for performance of proficiency panels for *Trichinella* should be included in this section. This section should refer to the QA recommendations and other guidance on proficiency panels as described in Section 3 of this document.

#### 4.3.9 Establishing a *Trichinella* testing laboratory

Testing for *Trichinella* in food animals and game meats intended for human consumption should be performed in a certified (or otherwise qualified) laboratory to ensure adequate QA. A description of the minimum requirements for laboratory certification is provided in Section 5 of these recommendations.

#### 4.3.10 Laboratory safety

An adequate description of the hazards, precautions and mitigations for working with *Trichinella*- infected meat should be described, including the use of Safety Data Sheets (SDS) as appropriate. This section should also describe the safe handling of reagents used in digestion testing including HCl and pepsin.

## 5 Recommendations for a *Trichinella* testing laboratory certification program

This section describes a set of essential components and minimum requirements for recognizing the competence of a laboratory program for *Trichinella* digestion testing. Although certification is the term used in this document to denote this recognition, other designations may also be appropriate. For the purpose of this document, the competent authority granting this recognition is considered the certifying body, and testing laboratories are those certified under the program (Forbes et al., 2005). The certifying body may designate a National Reference Laboratory or similarly qualified body to fulfill some or all of its responsibilities.

Laboratories conducting *Trichinella* testing should have a functional QMS with policies and procedures which incorporate QA, quality control, analyst competence, suitable facilities, validated method(s), and sample identification and traceability. The OIE (2018e) and ISO (2017) provide extensive recommendations and standards, respectively, for quality management in veterinary testing laboratories. Based on the same principles and guidelines, ten essential components are described in the remainder of this section and recommended by ICT. The minimum requirements to be met for each are recommended by ICT for a *Trichinella* testing laboratory certification program. Although some countries may not have sufficient veterinary infrastructure, regulatory oversight, or resources to fulfill all recommendations set forth here, this document should still serve as a useful reference.

### 5.1 Quality management system

A QMS is the foundation of any certification program and includes all policies, procedures and associated documentation required by both the certifying body and testing laboratory to ensure that testing is reliable and fit for the intended purpose. The specific policies and procedures should be described in a quality manual (or similar documentation of the QMS) or in supporting documents.

The certifying body should be third party- accredited by the national accreditation body or other signatory of the International Laboratory Accreditation Cooperation (ILAC) in accordance with the ISO/IEC 17025 standard for QA, with the *Trichinella* digestion assay included within its scope of accredited tests. The certifying body should similarly use QA in all prescribed policies, procedures and documentation pertaining to the delivery of the program, including training, proficiency sample provision, etc., as applicable.

It is also preferable that the testing laboratory be accredited to the ISO/IEC 17025 standard. The testing laboratory must, at a minimum, meet management system and technical requirements based on the principles of ISO/IEC 17025, and have a QMS approved by the certifying body.

### 5.2 Regulatory oversight

The certifying body should have the legally empowered authority to oversee and/or govern the certification process, and ultimately provides or approves all policies and procedures required for certified testing, including granting or revoking certification status of testing laboratories. This role would typically be held by the competent authority, veterinary authority, or similar authority for a particular country, as defined by OIE (2018f) and Codex Alimentarius Commission (CAC), 2005, Codex Alimentarius Commission (CAC), 2015, which provide international guidelines for the control of *Trichinella*. The extent of oversight required by the certifying body may vary based on the testing laboratory's level of QA recognition and the purpose of testing.

### 5.3 Program structure

A documented description of the certification program should be provided by the certifying body which clearly outlines the program organization and objectives (including purpose of testing) and respective roles and responsibilities of the certifying body and testing laboratory. This should include all requirements that must be met by the testing laboratory to achieve and maintain certification status, including any associated fees, legal agreements, reporting relationships, subcontracting, and regulatory obligations for suspect or positive test results. Key information on the program can be compiled into an information package provided to candidate laboratories, including a checklist of important milestones in the certification process, with expected time frames and assigned responsibilities (certifying body and/or testing laboratory) for each.

It is important that any laboratory certification program remain current with changes in knowledge, technology, and QA requirements pertaining to the purpose of testing, so that test reliability is not compromised. In order to achieve this, a thorough review of the program should be conducted periodically by the certifying body.

### 5.4 Standardized and validated assay

An assay with statistically sound validation data to scientifically demonstrate its fitness for intended purpose should be used, and is a requirement for any testing conducted within the scope of ISO/IEC 17025 accreditation. The magnetic stirrer method for pooled sample digestion has been extensively validated and is recommended by the ICT as the most reliable method for the detection of *Trichinella* larvae, and is also prescribed by the OIE and European Union (European Union, 2015; OIE, 2018a). Essential QA standards for digestion testing to ensure accuracy and reliability when performing this assay are provided in Section 2.

Depending on the purpose of testing and client demands (for example, to meet *Trichinella* testing requirements of importing countries) an alternate method may be stipulated for certified testing which is not equivalent to the recommended or prescribed method and which may not have been validated. In such cases there may be a requirement for bilateral agreement between the certifying body and client that the method is fit for the intended purpose. However, in the absence of adequate supporting QA data, the results from such tests cannot be considered reliable for food safety purposes.

### 5.5 Laboratory facilities and equipment

The minimum physical standards and specifications for certified testing should be provided by the certifying body. Biosafety Level 2 guidelines should be followed. Guidelines for facilities and equipment for basic (Biosafety Level 1 and 2) laboratories are provided in the Laboratory biosafety manual of the World Health Organization (WHO, 2004). The purpose of testing may also dictate other requirements such as situating the laboratory on the same premises as the slaughterhouse generating the carcasses for testing.

Recommended equipment with minimum specifications for performance of the digestion assay are included in Section 2. The extent of equipment required will vary according to anticipated sample load, number of staff, work hours and other factors related to each laboratory site.

### 5.6 Sample collection and handling

The certifying body should provide or approve all requirements for sampling, which may vary depending on purpose of testing, species tested, and client demands. Specific recommendations for sampling for the digestion assay are provided in Section 2. A detailed prescriptive SOP for sample collection and handling should be provided or approved by the certifying body, including any stipulations for sample identification, fitness criteria, storage, and decontamination and disposal.

### 5.7 Traceability

The certifying body should provide or approve identification and traceability procedures and associated documentation to reliably and uniquely link samples, test results and carcasses of origin, as well as procedures required when positive results are obtained, including any subsequent sample collection and testing to determine affected carcass(es), and the decontamination and disposal of positive carcasses.

If testing is done for food safety, disease control, or export purposes, carcasses should not leave the premises until negative test results have been obtained. This should entail procedures for detaining intact or processed carcasses, as well as for maintaining control of by-product and offal, pending negative test results, to prevent distribution of infected material.

The certifying body or other appropriate regulatory authority should have an effective mechanism, with requisite SOPs, for trace-back of any positive carcasses, using information provided by the slaughter facility and/or testing laboratory.

For domestic monitoring or surveillance purposes in non-endemic areas, the certifying body may lessen the above requirements based on assessment of risk, and the practicality and reliability of established procedures to enable traceability of any positive results to herd of origin.

### 5.8 Training

Technical training is necessary to ensure competency of analysts and that accurate and repeatable results are achieved by the laboratory performing the assay. Specific recommendations for training and qualifying analysts, and for contents of a training manual, are provided in Section 4. Training should be administered by a qualified provider, as determined by the certifying body. In addition to training in the performance of the digestion assay, analysts must be familiar with basic laboratory procedures and equipment, and with the QMS and safety policies and procedures of the testing laboratory. It is recommended that eligible candidate analysts successfully complete a standardized and documented training session to demonstrate competence prior to conducting certified testing, as described in Section 4.

### 5.9 Proficiency assessment

A reliable, standardized, validated and quality assured system to prepare and provide *Trichinella* proficiency samples is required for training, and for the ongoing assessment of competency of analysts and the testing laboratory quality system. Examples of such proficiency sample systems for *Trichinella* used by National Reference Laboratories have been published. Proficiency testing providers should be ISO/IEC 17025-accredited and/or preferably accredited to the ISO/IEC 17043 standard specific to PT (ISO, 2017; ISO, 2010). Specific recommendations to ensure QA in PT are provided in Section 3.

### 5.10 Audits

To ensure that all requirements of the certification program are met, a formal system of scheduled on-site audits of the testing laboratory is required to identify and document deficiencies in the QMS for testing, specify corrective actions, and effect satisfactory resolution. Such on-site assessments are also an opportunity to foresee potential technical needs and problems and implement continual improvements.

Scheduled internal on-site audits, conducted at least once annually, should be part of the QMS of the testing laboratory and required by the certifying body. Internal audits should be conducted by the testing laboratory's QA Manager (or similarly qualified personnel) or designate. Scheduled external on-site audits should also be conducted by the certifying body at least once biannually and/or by a third party such as the national accrediting body (for ISO/IEC 17025-accredited testing laboratories) at the prescribed frequency.

The certifying body must be kept informed of all internal or third party audit findings. Audit frequency may be increased based on previous audit findings, operational changes, proficiency failures, and ongoing corrective actions.

A standardized audit checklist should be used to ensure all aspects of the QMS for *Trichinella* testing are reviewed. Key elements should include facilities, personnel, training, equipment, quality manual (or similar), SOPs and record keeping.

The certifying body must have a standardized and transparent process for any corrective action requirements and commensurate deadlines to be met by the testing laboratory, and for the delay, suspension or revocation of certification status of the testing laboratory.

## 6 Conclusion

The information presented here represents current science-based recommendations of the ICT on QA practices related to the various aspects of *Trichinella* digestion testing programs. The ICT will update these recommendations as necessary to address relevant advances in science and technology. *Trichinella* testing laboratories that follow the recommendations in this document should be able to generate reliable test results and attain international recognition for quality assured testing. Updates will be made available on the ICT website at http://www.trichinellosis.org/.

The following are the supplementary data related to this article.

Appendix A ICT Quality Assurance Committee Members. Appendix A

Appendix B Definitions of QA terms applicable to digestion testing for *Trichinella*. Appendix B

## Conflicts of interest

The authors have no conflicts of interest.

## References

[bb0005] Alban L., Pozio E., Boes J., Boireau P., Boue F., Claes M., Cook A.J., Dorny P., Enemark H.L., van der Giessen J., Hunt K.R., Howell M., Kirjusina M., Noeckler K., Rossi P., Smith G.C., Snow L., Taylor M.A., Theodoropoulos G., Vallee I., Viera-Pinto M.M., Zimmer I.A. (2011). Towards a standardised surveillance for *Trichinella* in the European Union. Prev. Vet. Med..

[bb0010] Codex Alimentarius Commission (CAC) (2005). The Code of Hygienic Practice for Meat, Section 3.

[bb0015] Codex Alimentarius Commission (CAC) (2015). Guidelines for the Control of *Trichinella* spp. in Meat of Suidae. CAC/GL 86–2015. http://www.fao.org/fao-who-codexalimentarius/committees-and-task-forces/en/?provide=committeeDetail&idList=10.

[bb0020] Dupouy-Camet J., Bruschi F., Dupouy-Camet J., Murrell K.D. (2007). Management and diagnosis of human trichinellosis. FAO/WHO/OIE Guidelines for the Surveillance, Management, Prevention and Control of Trichinellosis.

[bb0025] European Union (2015). Commission implementing regulation (EU) 2015/1375 of 10 August 2015 laying down specific rules on official controls for *Trichinella* in meat. Off. J. EU.

[bb0030] European Union Reference Laboratory for Parasites (2019). PT: “Magnetic stirrer method for pooled sample digestion according to the regulation (EU) 2015/1375”. http://www.iss.it.

[bb0035] Forbes L.B., Gajadhar A.A. (1999). A validated *Trichinella* digestion assay and an associated sampling and quality assurance system for use in testing pork and horsemeat. J. Food Prot..

[bb0040] Forbes L.B., Rajic A., Gajadhar A.A. (1998). Proficiency samples for quality assurance in *Trichinella* digestion tests. J. Food Prot..

[bb0045] Forbes L.B., Parker S.E., Scandrett W.B. (2003). Comparison of a modified digestion assay with trichinoscopy for the detection of *Trichinella* larvae in pork. J. Food Prot..

[bb0050] Forbes L.B., Scandrett W.B., Gajadhar A.A. (2005). A program to accredit laboratories for reliable testing of pork and horse meat for *Trichinella*. Vet. Parasitol..

[bb0055] Gajadhar A.A., Forbes L.B. (2002). An internationally recognized quality assurance system for diagnostic parasitology in animal health and food safety, with example data on trichinellosis. Vet. Parasitol..

[bb0060] Gajadhar A.A., Pozio E., Gamble H.R., Nockler K., Maddox-Hyttel C., Forbes L.B., Vallée I., Rossi P., Marinculić A., Boireau P. (2009). *Trichinella* diagnostics and control: mandatory and best practices for ensuring food safety. Vet. Parasitol..

[bb0065] Gamble H.R. (1996). Detection of trichinellosis in pigs by artificial digestion and enzyme immunoassay. J. Food Prot..

[bb0070] Gamble H.R., Bessonov A.S., Cuperlovic K., Gajadhar A.A., Knapen F. Van, Nockler K., Schenone H., Zhu X. (2000). International Commission on Trichinellosis: recommendations on methods for the control of *Trichinella* in domestic and wild animals intended for human consumption. Vet. Parasitol..

[bb0075] Gamble H.R., Boireau P., Nockler K., Kapel C.M.O., Dupouy-Camet J., Murrell K.D. (2007). Prevention of *Trichinella* infection in the domestic pig. FAO/WHO/OIE Guidelines for the Surveillance, Management, Prevention and Control of Trichinellosis, Paris.

[bb0080] Gottstein B., Pozio E., Nöckler K. (2009). Epidemiology, diagnosis, treatment, and control of Trichinellosis. Clin. Microbiol. Rev..

[bb0085] International Air Transport Association (IATA) (2019). UN 3373. Dangerous Goods Regulations.

[bb0100] International Organisation for Standardisation (ISO) ISO 18743 (2015). Microbiology of the Food Chain - Detection of Trichinella Larvae in Meat by Artificial Digestion Method.

[bb0095] International Organisation for Standardisation (ISO) ISO 13528 (2015). Statistical Methods for Use in Proficiency Testing by Interlaboratory Comparisons.

[bb0105] International Organisation for Standardisation (ISO) ISO 22117 (2019). Microbiology of the Food Chain- Specific Requirements and Guidance for Proficiency Testing by Interlaboratory Comparison.

[bb0110] International Organisation for Standardisation (ISO) ISO/IEC 17025 (2017). General Requirements for the Competence of Testing and Calibration Laboratories.

[bb0115] International Organisation for Standardisation (ISO) ISO/IEC 17043 (2010). Conformity Assessment – General Requirements for Proficiency Testing.

[bb0120] Kapel C.M.O., Measures L., Møller L.N., Forbes L., Gajadhar A. (2003). Experimental *Trichinella* infection in seals. Int. J. Parasitol..

[bb0125] Kapel C.M.O., Webster P., Gamble R. (2005). Muscle distribution of sylvatic and domestic *Trichinella* larvae in production animals and wildlife. Vet. Parasitol..

[bb0130] Larter N.C., Forbes L.B., Elkin B.T., Allaire D.G. (2011). Prevalence of *Trichinella* spp. in black bears, grizzly bears, and wolves in the Dehcho Region, Northwest Territories, Canada, including the first report of *T. nativa* in a grizzly bear from Canada. J. Wildl. Dis..

[bb0135] Leclair D., Forbes L.B., Suppa S., Gajadhar A.A. (2003). Evaluation of a digestion assay and determination of sample size and tissue for the reliable detection of *Trichinella* larvae in walrus meat. J. Vet. Diagn. Investig..

[bb0140] Malakauskas A., Paulauskas V., Keidans P., Järvis T., Eddi C., Kapel C.M.O. (2007). Molecular epidemiology of *Trichinella* spp. in the three Baltic countries: Lithuania, Latvia and Estonia. Parasitol. Res..

[bb0145] Marucci G., Pezzotti P., Pozio E. (2009). Ring trial among National Reference Laboratories for parasites to detect *Trichinella spiralis* larvae in pork samples according to the EU directive 2075/2005. Vet. Parasitol..

[bb0150] Murrell K.D., Pozio E. (2011). Worldwide occurrence and impact of human trichinellosis, 1986-2009. Emerg. Infect. Dis..

[bb0155] Nöckler K., Kapel C.M.O., Dupouy-Camet J., Murrell K.D. (2007). Detection of *Trichinella*, meat inspection and hygiene, legislation. FAO/WHO/OIE Guidelines for the Surveillance, Management, Prevention and Control of Trichinellosis.

[bb0160] Nöckler K., Pozio E., Voigt W.P., Heidrich J. (2000). Detection of *Trichinella* infection in food animals. Vet. Parasitol..

[bb0165] Nöckler K., Reckinger S., Szabo I., Maddox-Hyttel C., Pozio E., van der Giessen J., Vallée I., Boireau P. (2009). Comparison of three artificial digestion methods for detection of non-encapsulated *Trichinella pseudospiralis* larvae in pork. Vet. Parasitol..

[bb0170] Pozio E., Dupouy-Camet J., Murrell K.D. (2007). Taxonomy, biology and epidemiology of *Trichinella* parasites. FAO/WHO/OIE Guidelines for the Surveillance, Management, Prevention and Control of Trichinellosis.

[bb0175] Pozio E., Zarlenga D. (2019). International Commission on Trichinellosis: recommendations for genotyping *Trichinella* muscle stage larvae. Food and Waterborne Parasitology.

[bb0180] Special Issue (2000). *Trichinella* and Trichinellosis. Vet. Parasitol..

[bb0185] USDA (2018). FSIS Compliance Guideline for the Prevention and Control of *Trichinella* and Other Parasitic Hazards in Pork Products. https://www.fsis.usda.gov/wps/wcm/connect/b596a9b8-c3fb-4529-8395-8aacc493a9fd/Compliance-Guidelines-Trichinella.pdf?MOD=AJPERES.

[bb0190] Vallée I., Mace P., Forbes L., Scandrett B., Durand B., Gajadhar A., Boireau P. (2007). The use of proficiency samples to assess diagnostic laboratories in France performing a *Trichinella* digestion assay. J. Food Prot..

[bb0195] Webster P., Maddox-Hyttel C., Nöckler K., Malakauskas A., van der Giessen J., Pozio E., Boireau P., Kapel C.M.O. (2006). Meat inspection for *Trichinella* in pork, horsemeat and game within EU -available technology and its present implementation. Eurosurveillance.

[bb0200] World Health Organization (WHO) (2004). Laboratory Biosafety Manual.

[bb0210] World Organisation for Animal Health (OIE) (2018). Trichinellosis (infection with *Trichinella* spp.). Manual of Diagnostic Tests and Vaccines for Terrestrial Animals.

[bb0215] World Organisation for Animal Health (OIE) (2018). Infection with *Trichinella* spp.. Terrestrial Animal Health Code, Chapter 8.17.

[bb0220] World Organisation for Animal Health (OIE) (2018). Collection, submission and storage of diagnostic specimens. Manual of Diagnostic Tests and Vaccines for Terrestrial Animals, Chapter 1.1.2.

[bb0225] World Organisation for Animal Health (OIE) (2018). Transport of biological materials. Manual of Diagnostic Tests and Vaccines for Terrestrial Animals.

[bb0230] World Organisation for Animal Health (OIE) (2018). Quality management in veterinary testing laboratories. Manual of Diagnostic Tests and Vaccines for Terrestrial Animals. Chapter 1.1.5.

[bb0235] World Organisation for Animal Health (OIE) (2018). Glossary. Terrestrial Animal Health Code.

