# Supplementary material for: International Commission on Trichinellosis: Recommendations for quality assurance in digestion testing programs for Trichinella
Source: Food Waterborne Parasitol. 2019 Jun 5;16:e00059. doi: 10.1016/j.fawpar.2019.e00059 (PMC7033998; doi:10.1016/j.fawpar.2019.e00059)
Supplement: Appendix A — ICT Quality Assurance Committee Members. [file mmc1.docx]

Supplemental Data

APPENDIX A. ICT Quality Assurance Committee Members

**Committee on QA in Digestion Testing Programs for *Trichinella***

Alvin Gajadhar Canada Committee Chair

**Sub-Committee on QA for Digestion Testing**

Karsten Noeckler Germany Group Leader

Christian Kapel Denmark Member

**Sub-Committee on QA for Proficiency Panels**

Pascal Boireau France Proficiency Sample Production Group Leader

Marleen Claes Belgium Member

Patrizia Rossi Italy Proficiency Testing Panels Group Leader

Sandrine Lacour France Member

Frits Franssen Netherlands Member

Lorry Forbes Canada Proficiency Testing Evaluation Group Leader

Edoardo Pozio Italy Member

Isabelle Vallée France Member

**Sub-Committee on QA for Laboratory Certification**

Brad Scandrett Canada Group Leader

Clive Pigott New Zealand Member

Edoardo Pozio Italy Member

**Sub-Committee on QA for Technician Training**

Ray Gamble USA Group Leader

Bruno Gottstein Switzerland Member

**Other Committee Members**

Francisco Bolas Spain

Jean Dupouy-Camet France

Caroline Frey Switzerland

Teresa Garate Spain

Joke van der Giessen Netherlands

Albert Marinculic Croatia

Liu Mingyuan China

Juan Olmedo Spain

Ljiljana Sofronic Serbia
