# Supplementary material for: International Commission on Trichinellosis: Recommendations for quality assurance in digestion testing programs for Trichinella
Source: Food Waterborne Parasitol. 2019 Jun 5;16:e00059. doi: 10.1016/j.fawpar.2019.e00059 (PMC7033998; doi:10.1016/j.fawpar.2019.e00059)
Supplement: Appendix B — Definitions of QA terms applicable to digestion testing for Trichinella. [file mmc2.doc]

Supplemental Data

APPENDIX B. Definitions of QA Terms Applicable

to Digestion Testing for *Trichinella*

| **Terms** | **Definition** |
| --- | --- |
| **Quality assurance** | A general term referring to all aspects of laboratory management, technical procedures and documentation which are used for ensuring that a test is fit for an intended purpose such as the detection of *Trichinella* larvae in meat for food safety |
| **Quality system** | A system of detailed management and laboratory procedures required to provide and document a *Trichinella* testing system that is fit for its intended purpose. Components of the Quality System are formally listed in the Quality Assurance Manual. |
| **Quality Manual** | The Quality Manual describes the laboratory’s quality management system (QMS). It defines the scope of the QMS and the policies to provide reasonable assurances of effective planning, operation, and control of processes for achieving the specified quality objectives. The *Trichinella* testing laboratory’s Quality Manual describes management activities and processes related to organization, personnel, equipment and facilities, purchasing and inventory, process control and improvement, documents, records, and information management, occurrence management, service and training and competence requirements. |
| **Quality control** | Required routine laboratory activities in the performance of a diagnostic test to ensure accurate, reliable and reproducible results. This includes documented evidence of strict adherence to Standard Operating Procedures for the specific test, including training, reagents, equipment, operating conditions, critical control points (CCPs), recording, and reporting. |
| **Proficiency testing**  **(Conformity assessment)** | Evaluation of technical competence in the performance of specific *Trichinella* test procedures. Results can be used to assess laboratory competence via interlaboratory comparisons, and/or the competence of individual analysts. Participating laboratories should use the same or equivalent test methods, reagents and controls. |
| **Proficiency sample** | A sample prepared for use in assessing technical competence and for training. It contains a known amount of the material to be measured such as *Trichinella* larvae in an appropriate meat matrix. |
| **Proficiency panel** | A set of proficiency samples used at a particular time for testing proficiency of analysts or for other purposes such as method validation and ring trials. |
| **Validation** | Establishment and verification of the performance characteristics of a test method using scientifically valid processes. Data produced by validation studies are used to demonstrate that a test is fit for its intended purpose and to defend its use. |
| **Standardization** | A physical or mathematical adjustment or correction of a measurement system to make the measurements conform to predetermined values. For digestion tests, this involves procedural modifications to ensure the required sensitivity, as measured by minimal acceptable detection of larvae in quality standard samples. |
| **Harmonization** | Agreement between laboratories to calibrate similar test methods, adjust diagnostic thresholds and express test data in such a manner as to allow uniform interpretation of results between laboratories or jurisdictions. |
| **Equivalency** | Test equivalency is relative to a reference method or another test, and is based on interlaboratory comparison of specific test performance characteristics. Participating laboratories should operate within an accredited quality system and use appropriate documentation, reagents and controls. |
| **Digestion assay** | A method to detect *Trichinella* larvae in muscle tissue that uses an enzymatic (pepsin-HCl) digestion step to release larvae from muscle tissue followed by concentration and microscopy steps for larval detection. |
| **Parasite identification** | Morphological, immunological and/or molecular identification of *Trichinella* spp. |
| **Critical control point (CCP)** | A step in the method, procedure, test, assay or analysis for which any deviation from a specified range of values could significantly affect test results. For example, digestion time and temperature are among the critical control points in digestion assays. |
| **Accuracy** | Level of agreement between a test value and the expected value for a reference standard. |
| **Precision** | Degree of dispersion of results for a repeatedly tested sample or in the case of digestion tests for detection of larvae in multiple reference standards. |
| **Repeatability** | Level of agreement between replicates of a sample both within and between runs of the same test method in a given laboratory, or in the case of digestion tests, the level of agreement for detection of larvae per gram of tissue within and between runs of the same test method in a given laboratory. |
| **Reproducibility** | Ability of a test method to provide consistent results when applied to aliquots of the same sample tested at different laboratories or in the case of digestion tests consistent results for detection of larvae in proficiency samples. |
| **Sensitivity** | *Analytical*: The lowest number of *Trichinella* larvae consistently detected by a digestion test.  *Diagnostic*: Proportion of known infected animals or known infected samples that test positive in an assay; known infected animals or known infected samples that test negative are considered to have false-negative results. |
| **Specificity** | *Analytical*: The degree to which the digestion test distinguishes between *Trichinella* (at the genus level) and non-targeted worms or artefacts.  *Diagnostic*: Proportion of known non-infected reference animals or non-infected samples that test negative in the assay; non-infected reference animals or non-infected samples that test positive are considered to have false-positive results. |
| **Standard operating procedure (SOP)** | A documented procedure that clearly defines the purpose of a method or test and all technical requirements for accurate, precise, repeatable and reproducible performance of the procedure. Elements of an SOP should include an explanatory narrative, detailed equipment and reagent list, critical control points, technical notes, performance instructions and directions for record keeping. |
| **Prescribed test method** | A method that is stipulated and required for a particular use, such as trade, by a regulatory organization (OIE, EU) or an individual country. It is not necessarily the most effective method. Often referred to as a standard method. |
| **Standard test method (OIE)** | A test method described in the OIE Manual of Standards for Diagnostic Tests and Vaccines which is designated as a prescribed test for international trade. |
| **Reference test method** | A method for use as a benchmark or gold standard. It is not necessarily the most effective method. Its primary function is to provide a common basis for the comparison of both new and existing methods. |
| **ICT-recommended test method** | A method supported by the ICT that has satisfactory validation data to demonstrate that it is fit for its intended purpose. An ICT-recommended method is a candidate for use as a prescribed method, standard method reference method or alternative method. |
| **Alternative method** | A method that is authorized for use instead of a standard, prescribed or reference method based on fitness for purpose for local situations. This may be a non-equivalent method or a method with insufficient validation data and can only be used for import/export with bilateral agreement. |
| **Screening method** | A method which serves to screen samples to determine if a sample may be positive and whether further testing is required. For digestion assays this could be batch testing of pooled samples representing a number of animals. |
| **Confirmatory method** | A method to definitively confirm the infection of a sample or animal, and which could involve more than one assay or procedure. For digestion assays, this could be the same method as used for screening pooled samples, but this time used for individual samples from the initial pool, or it could comprise a multifaceted approach such as follow-up serological and digestion testing of the herd of origin. |
| **Laboratory manager** | Responsible for managing the overall aspects of the laboratory including administration, human resources, budget, quality assurance, training and safety. |
| **Quality manager** | Responsible for designing, maintaining, auditing and reporting on all aspects of the quality system. |
| **Laboratory supervisor** | Responsible for the day to day operation of a testing laboratory for one or more tests, including adherence to quality system requirements to provide quality control. |
| **Qualified analyst** | A technologist who is formally recognized as having the required knowledge, skills and proficiency required to perform a specific test method following training and formal evaluation. |
| **Reference laboratory** | A laboratory formally recognised by a national or international authority for a required level of scientific and diagnostic expertise for a particular animal disease and/or testing methodology; includes capability for characterising and assigning values to reference reagents and samples and provision of training and audits. |
| **Certified laboratory** | A laboratory that is formally recognized by a government agency for competency in performing a prescribed diagnostic test or tests. Although this may have a significant quality assurance component, certification does not imply that the laboratory meets ISO/IEC 17025 quality system requirements. |
| **Accredited laboratory** | A laboratory meeting ISO/IEC 17025 quality system requirements as determined by a national or international third party auditor. |
| **Competence** | Overall measure of ability to perform specific tests in a consistent and quality manner; includes in-house checks and interlaboratory comparisons. |

**References Related to QA Terms:**

International Organisation for Standardisation (ISO). ISO/IEC 17000: 2004. Conformity assessment – Vocabulary and general principles. Geneva, Switzerland.

International Organisation for Standardisation (ISO). ISO/IEC Guide 99: 2007. International vocabulary of metrology – Basic and general concepts and associated terms (VIM). Geneva, Switzerland.

International Organisation for Standardisation (ISO). ISO/IEC 17043:2010. Conformity Assessment – General requirements for proficiency testing. Geneva, Switzerland.

International Organisation for Standardisation (ISO). ISO/IEC 17025:2017. General requirements for the competence of testing and calibration laboratories. Geneva, Switzerland.

World Organisation for Animal Health (OIE). 2008. OIE Standard for Management and Technical Requirements for Laboratories Conducting Tests for Infectious Animal Diseases *In*: OIE Quality Standard and Guidelines for Veterinary Laboratories: Infectious diseases. OIE, 12 rue de Prony, 75017 Paris, France, 1-31.
